# Folate Receptor Alpha: A New Tool in the Diagnosis and Treatment of Lung Cancer

**DOI:** 10.18632/oncotarget.575

**Published:** 2012-07-24

**Authors:** Laura D. Wood

**Affiliations:** Department of Pathology, Johns Hopkins University School of Medicine, Baltimore, MD, USA

Identification of specific molecular alterations in cancer has revolutionized the diagnosis and treatment of the disease. Study of these alterations has not only greatly expanded our understanding of the biology of carcinogenesis – perhaps more importantly, these molecular alterations have been developed for clinically relevant applications, such as diagnostic tests, prognostic markers, and targets for rational therapeutic approaches. With the recent explosion of genomic, transcriptomic, and proteomic data from a wide variety of human cancers, the use of these molecular alterations as clinical tools will continue to rapidly expand. However, despite these rapid advances, each alteration must still be carefully validated to ensure that its clinical development is justified and will lead to an advance that improves the diagnosis and treatment of cancer.

In a recent issue of *Oncotarget*, O'Shannessy *et al* described important steps in the development of folate receptor alpha (FRA) expression as one such clinical tool [1]. This cell surface glycoprotein can mediate the transport of folate into cells, though it is an uncommon mechanism of folate transport in adult tissues – thus, it is expressed on the apical surface of only a subset of normal and neoplastic cells [2]. FRA is highly expressed in both lung and ovarian carcinomas, and expression levels have been correlated with prognosis in both tumor types [3,4]. In the recent *Oncotarget* study, the authors characterized an immunohistochemical assay with a specific monoclonal antibody for the FRA protein, confirming its efficacy in both formalin-fixed paraffin-embedded tissue sections as well as fine needle aspiration specimens. In addition to analyzing its expression in a variety of normal tissues, the authors focused on its expression in lung cancers – they reported that immunohistochemical labeling for FRA was positive in a large proportion of lung adenocarcinomas, while it was negative in most squamous cell carcinomas. Thus, FRA expression is an additional characteristic that distinguishes adenocarcinoma from squamous cell carcinoma, and further investigation of the molecular basis of this difference may provide additional insights into the distinct behavior of these two cancer types. In addition, immunohistochemical labeling for FRA may also possess clinical utility in that it could add discriminatory power to the current routine panel for the work-up of lung cancer. However, further studies will be necessary to assess the diagnostic benefit of the addition of FRA to currently employed immunohistochemical stains in this differential diagnosis.

The authors also took a further step to expand the clinical applicability of their findings – they showed that patients with lung adenocarcinomas with high FRA expression (as assessed by their immunohistochemical assay) had improved survival compared to those with low FRA expression. Although the association of FRA expression and survival in lung cancer has been previously reported using mRNA-based RT-PCR assays [3], this study represents the first association of survival and FRA protein expression. As immunohistochemistry is widely available in most pathology laboratories and is far less expensive than molecular studies, this finding is an important advance in the clinical adoption of FRA expression analysis. Thus, routine immunohistochemical analysis of FRA expression may provide additional prognostic information for patients with lung adenocarcinoma, as the survival benefit was significant even after adjusting for stage, age, gender, and race.

Folate receptors are promising targets for novel therapeutics, and several therapies directed against FRA have been developed. A monoclonal antibody targeting FRA inhibits the growth of cancer cells overexpressing FRA *in vitro* as well as in mouse xenografts, and the antibody (now called Farletuzumab) is currently in clinical trials in human cancer patients [5,6]. The described immunohistochemical assay could play a crucial role in identifying patients likely to benefit from this novel therapy, as preclinical studies show growth inhibition in cells overexpressing FRA. However, the correlation of FRA expression (as assayed by immunohistochemistry) and response to FRA-targeted therapy must be specifically confirmed in human cancer patients before this assay is widely used to determine eligibility for clinical trials.

In their study of FRA in lung adenocarcinoma, O'Shannessy *et al* employed rigorous validation of a potentially useful clinical tool – using an immunohistochemical assay with a specific monoclonal antibody, they show that FRA expression is relatively specific for lung adenocarcinoma and that high FRA expression is associated with improved survival. Though further work will be necessary to translate this assay (and the related targeted therapy) into human cancer patients, their study confirmed the potential of FRA as a prognostic marker in lung adenocarcinoma.
